# A randomised, open-label, parallel group phase 2 study of antisense oligonucleotide therapy in acromegaly

**DOI:** 10.1530/EJE-18-0138

**Published:** 2018-05-22

**Authors:** Peter J Trainer, John D C Newell-Price, John Ayuk, Simon J B Aylwin, Aled Rees, William Drake, Philippe Chanson, Thierry Brue, Susan M Webb, Carmen Fajardo, Javier Aller, Ann I McCormack, David J Torpy, George Tachas, Lynne Atley, David Ryder, Martin Bidlingmaier

**Affiliations:** 1 Department of EndocrinologyThe Christie NHS Foundation Trust, University of Manchester, Manchester Academic Health Science Centre, Manchester, UK; 2 Department of Oncology and MetabolismThe Medical School, University of Sheffield, Sheffield, UK; 3 Royal Hallamshire HospitalSheffield Teaching Hospitals NHS Foundation Trust, Sheffield, UK; 4 Medicine EndocrinologyQueen Elizabeth Hospital Birmingham, Edgbaston, UK; 5 King’s College HospitalLondon, UK; 6 Neuroscience and Mental Health Research InstituteSchool of Medicine, Cardiff University, Hadyn Ellis Building, Cardiff, UK; 7 Department of EndocrinologySt Bartholomew’s Hospital, London, UK; 8 Assistance Publique-Hôpitaux de ParisHôpitaux Universitaires Paris-Sud, Hôpital de Bicêtre, Service d’Endocrinologie et des Maladies de la Reproduction, Le Kremlin-Bicêtre, France; 9 Inserm 1185Fac Med Paris Sud, Univ Paris-Sud, Université Paris-Saclay, Le Kremlin-Bicêtre, France; 10 Aix-Marseille UniversitéCNRS, CRN2M UMR 7286, Marseille, France; 11 APHMHôpital Conception, Service d’Endocrinologie, Diabète et Maladies Métaboliques, Centre de Référence des Maladies Rares d’Origine Hypophysaire, Marseille, France; 12 Department of EndocrinologyCIBERER Group 747, IIB-S Pau, Hospital de la Santa Creu i Sant Pau, Universitat Autonoma de Barcelona, Barcelona, Spain; 13 Servicio de EndocrinologíaHospital Universitario de La Ribera, Alzira, Valencia, Spain; 14 Endocrinology DepartmentHospital Universitario Puerta de Hierro Majadahonda, Majadahonda, Spain; 15 Garvan Institute of Medical Research and St Vincent’s HospitalDarlinghurst Sydney, New South Wales, Australia; 16 Royal Adelaide HospitalNorth Terrace, Adelaide, Australia; 17 Antisense Therapeutics LimitedToorak, Victoria, Australia; 18 Manchester Academic Health Science Centre (MAHSC) Clinical Trials UnitThe Christie NHS Foundation Trust, University of Manchester, Manchester, UK; 19 Endocrine LaboratoryMedizinische Klinik und Poliklinik IV, Klinikum der Ludwig-Maximilians-Universität München, Munich, Germany

## Abstract

**Objective:**

ATL1103 is a second-generation antisense oligomer targeting the human growth hormone (GH) receptor. This phase 2 randomised, open-label, parallel-group study assessed the potential of ATL1103 as a treatment for acromegaly.

**Design:**

Twenty-six patients with active acromegaly (IGF-I >130% upper limit of normal) were randomised to subcutaneous ATL1103 200 mg either once or twice weekly for 13 weeks and monitored for a further 8-week washout period.

**Methods:**

The primary efficacy measures were change in IGF-I at week 14, compared to baseline and between cohorts. For secondary endpoints (IGFBP3, acid labile subunit (ALS), GH, growth hormone-binding protein (GHBP)), comparison was between baseline and week 14. Safety was assessed by reported adverse events.

**Results and conclusions:**

Baseline median IGF-I was 447 and 649 ng/mL in the once- and twice-weekly groups respectively. Compared to baseline, at week 14, twice-weekly ATL1103 resulted in a median fall in IGF-I of 27.8% (*P* = 0.0002). Between cohort comparison at week 14 demonstrated the median fall in IGF-I to be 25.8% (*P* = 0.0012) greater with twice-weekly dosing. In the twice-weekly cohort, IGF-I was still declining at week 14, and remained lower at week 21 than at baseline by a median of 18.7% (*P* = 0.0005). Compared to baseline, by week 14, IGFBP3 and ALS had declined by a median of 8.9% (*P* = 0.027) and 16.7% (*P* = 0.017) with twice-weekly ATL1103; GH had increased by a median of 46% at week 14 (*P* = 0.001). IGFBP3, ALS and GH did not change with weekly ATL1103. GHBP fell by a median of 23.6% and 48.8% in the once- and twice-weekly cohorts (*P* = 0.027 and *P* = 0.005) respectively. ATL1103 was well tolerated, although 84.6% of patients experienced mild-to-moderate injection-site reactions. This study provides proof of concept that ATL1103 is able to significantly lower IGF-I in patients with acromegaly.

## Introduction

Acromegaly is a rare, chronic, life-shortening disease caused by hypersecretion of growth hormone (GH), virtually always due to a pituitary adenoma, that in turn results in elevated circulating levels of insulin-like growth factor 1 (IGF-I) (1). Conventional therapy is directed at the pituitary gland and attempts to reduce GH secretion by means of surgery, radiotherapy or medical therapy in the form of somatostatin analogues and dopamine agonists (2). The GH receptor antagonist pegvisomant has successfully exploited an alternative therapeutic approach, namely to block GH action rather than secretion (3).

Antisense oligonucleotides (ASOs) are single-stranded synthetic oligonucleotides that have been developed as therapeutic agents. Translation of mRNA, and hence, protein synthesis, is inhibited by sequence-specific ASOs that bind target pre-mRNA and/or mRNA (4). In the early 1990s, clinical trials with ASOs began, and in 1998, fomivirsen became the first oligonucleotide to be approved by the US Food and Drug Administration (FDA) for the treatment of cytomegalovirus retinitis (5). In 2013, the second-generation ASO inhibitor mipomersen was approved by the FDA for the treatment of homozygous familial hypercholesterolaemia. Currently, there are more than 30 second-generation ASOs, including ATL1103, in clinical development for a variety of neurological, oncological, cardiovascular and metabolic conditions. Excellent reviews of the technology are available elsewhere (6).

**Figure 1:**
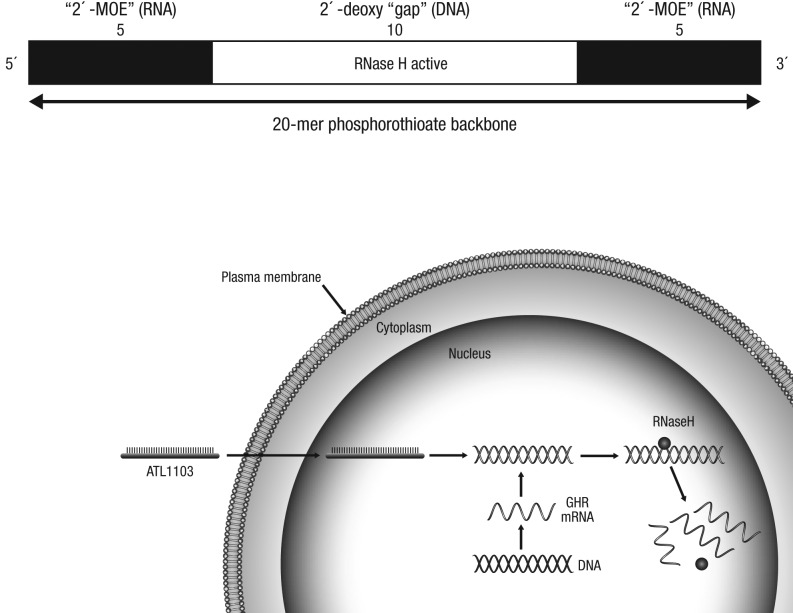
Cartoon representation of ATL1103 and the mechanism of antisense inhibition. ATL1103 is a second-generation, 20-mer antisense oligonucleotide with a phosphorothioate backbone and 2′-O-methoxyethyl modifications of the terminal five nucleotides at each end, which in combination increase its plasma half-life and affinity for the mRNA. Post-hybridisation RNase H degradation results in inhibition of GHR translation. GHR, growth hormone receptor.

ATL1103 is a second-generation, antisense oligomer designed to inhibit translation of human growth hormone receptor (*GHR*) mRNA (Fig. 1). It comprises 20 nucleotides with a phosphorothioate backbone and 2′-O-methoxyethyl modifications of the terminal five nucleotides at each end, which in combination increase its plasma half-life and affinity for the mRNA. Post-hybridisation RNase H degradation results in inhibition of GHR translation. In pre-clinical rodent and primate studies, ATL1103 reduced GHR mRNA levels in the liver and serum IGF-I, with a terminal half-life of 2–4 weeks (7). Phase 1 studies in healthy male volunteers demonstrated a fall in serum IGF-I and growth hormone–binding protein (GHBP) (https://www.asx.com.au/asxpdf/20111207/pdf/4234016x2cj5xn.pdf).

The objectives of this study were to evaluate the safety, tolerability and efficacy of ATL1103 in patients with acromegaly. Serum IGF-I was the primary measure of efficacy, with the other components of the IGF ternary complex, namely IGF-binding protein 3 (IGFBP3) and acid labile subunit (ALS) being additional measures of disease activity. Circulating GH and GHBP, the cleaved extracellular component of the GHR, were monitored to provide insight into the physiological implications of an ASO targeting the GHR. For the primary efficacy variable, the null hypothesis of no percentage change in fasting IGF-I levels from baseline to week 14 was tested for each treatment regimen.

## Subjects and methods

### Study design

**Figure 2:**
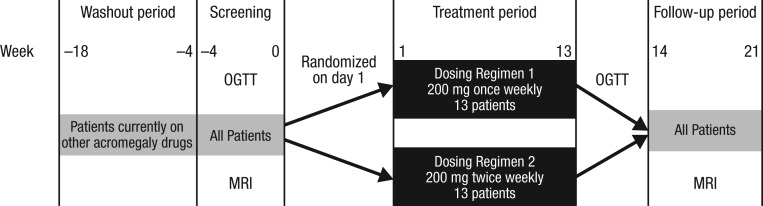
Schematic representation of study protocol. The protocol entailed appropriate washout from any ongoing acromegaly medical therapy after which serum IGF-I had to be at least >1.3 times age-related ULN. All patients underwent pituitary MRI scans at baseline and completion of the study drug. An OGTT was undertaken at baseline (after washout) and again at the end of week 13. IGF-I, insulin-like growth factor 1; OGTT, oral glucose tolerance test; ULN, upper limit of normal.

This was a phase 2 randomised, open-label, parallel-group study of the safety, tolerability, pharmacokinetics and efficacy of two subcutaneous dosing regimens of ATL1103 in patients with acromegaly (Fig. 2).

### Exclusion/inclusion criteria

#### Inclusion criteria

Patients who

1. provided written informed consent in accordance with local regulations,
2. were 18–80 years of age, inclusive,
3. had acromegaly due to pituitary adenoma (microadenoma or macroadenoma) identified by MRI,
4. had serum IGF-I level at screening >1.3 times the upper limit of normal (ULN),
5. had documented serum GH nadir levels >1 ng/mL at all test time points within the 2 h post oral glucose load for an oral glucose tolerance test (OGTT) (this could be historical),
6. were acromegaly treatment naïve or who had not taken other acromegaly medications for at least the following periods of time prior to IGF-I and GH screening tests: bromocriptine: 6 weeks; carbergoline: 8 weeks; quinagolide: 8 weeks; octreotide (subcutaneous): 4 weeks; pegvisomant: 8 weeks; octreotide LAR: 4 months; lanreotide (all presentations): 4 months,
7. had a BMI ≥19 kg/m^2^,
8. had adequate venous access to allow collection of multiple blood samples during the study,
9. were female of non–child-bearing potential (i.e., either surgically sterilised or at least 1 year post-menopausal), or, if of child-bearing potential, agreed to use two approved methods of contraception for the duration of the study and for 3 months after administration of the last dose of study drug, or were male and surgically sterilised or agreed to use an approved method of contraception for the duration of the study and for 3 months after administration of the last dose of study drug,
10. were willing and able to self-administer subcutaneous injections.

(Inclusion criteria 5, 6 and 9 were amended in protocol amendments during the study. For inclusion criterion 5, the requirement for GH after OGTT at screening was altered, as this could be historical. Inclusion criterion 6 was amended to clarify that the washout periods detailed were minimum periods. For inclusion criterion 9, contraceptive requirements were clarified.)

#### Exclusion criteria

Patients who

1. had acromegaly due to reasons other than pituitary adenoma,
2. had a pituitary adenoma that was less than 3 mm distance from the optic chiasm,
3. had undergone pituitary surgery within the 3 months preceding the screening visit,
4. had received pituitary radiotherapy within the 1 year preceding the baseline visit,
5. had insulin-treated diabetes or had commenced a new hypoglycaemic drug for diabetes within the 2 months prior to screening,
6. had congestive heart failure, unstable angina, clinically significant cardiac arrhythmia, or a history of acute myocardial infarction within the 3 months preceding the baseline visit,
7. had abnormal hepatic function at screening defined any of the following parameters >2× ULN: aspartate aminotransferase (AST), alanine aminotransferase (ALT), gamma glutamyl transferase (GGT), alkaline phosphatase (ALP), prothrombin time or total bilirubin,
8. had hepatitis B, hepatitis C or chronic liver disease,
9. were pregnant or lactating,
10. had known human immunodeficiency virus (HIV) (not tested specifically for this protocol) or history of immunodeficiency that may have compromised their safety or affected results from this study,
11. had a history of alcohol or drug abuse in the 6-month period preceding the baseline visit.

Patients were recruited in 13 tertiary referral centres in Australia, France, Spain and the United Kingdom.

The study was approved by the NRES Committee East Midlands, Derby, UK; Bicêtre Hospital, France; Ethical Committee of Clinical Research, Hospital del al Santa Crue I Sant Pau, Barcelona, Spain and Royal Adelaide Human Research Ethics Committee, Adelaide, Australia. The study was registered as EudraCT 201200314730 and ANZCTR 12611000854932. Patients gave written informed consent.

### Procedures and study medication

Patients received either ATL1103 200 mg once or twice weekly 3 and 4 days apart for 13 weeks, with every patient receiving three doses in the first week, administered every other day. Based on the tissue half-life of >4 weeks, experience from primate studies and data from the phase 1 study, additional ‘loading’ doses were administered in the first week.

ATL1103 is formulated as a ‘ready-to-inject’ sterile solution at a concentration of 200 mg/mL, pH 7.4, in ‘Water for Injection’. Patients were taught to self-administer ATL1103 subcutaneously. After completion of drug administration at the end of 13 weeks, patients were monitored, off all therapy for acromegaly, for a further 8 weeks.

All patients underwent pituitary MRI scans at baseline and at week 13 (completion of the study drug), which were independently reviewed by two ‘blinded’ expert pituitary neurosurgeons. Tumour diameter changes of 2 mm or more in any one dimension or tumour volume changes of more than 20% were considered significant.

An OGTT with measurement of plasma glucose and serum GH was undertaken at baseline (after any drug washout) and again at the end of week 13. An adverse event (AE) assessment was undertaken at each of the 11 study visits from baseline until study conclusion.

In addition to routine safety parameters, serum IGF-I, IGFBP3, ALS and GHBP were monitored. Ring size (fourth digit left hand) was measured using standard European-sized jewellers’ ring sets, and patients completed a signs and symptoms score (SSS, maximum score 40) and the disease-generated ‘quality of life’ AcroQol. AcroQol comprises 22 questions divided into two main categories: physical and psychological function. The psychological category is further subdivided into appearance and personal relationships. Each question is scored out of 5, with a maximum score of 110 reflecting best possible quality of life. The result is then converted to a percentage (8).

### Randomisation and blinding

Permuted block randomisation (generated by a statistician and imported into the electronic case report form) was used to assign patients to either open-label, once- or twice-weekly ATL1003. Once initial data for a patient had been entered and the patient had fulfilled all inclusion criteria, a randomisation number and treatment regimen were generated. Treatment allocation was not known to the operational personnel until this randomisation was performed.

Blocks of size four were used with no stratification for the first 24 patients. The list included an additional 24 randomisation numbers using a block size of 2 (total 48) to allow for overage.

### Assays

#### IGF-I, GH and IGFBP3

Serum IGF-I, GH and IGFBP3 were measured centrally by IDS-iSYS (Immunodiagnostic Systems, (IDS) Ltd., Boldon, England, UK) assays at the Endocrine Laboratory, Universität München (Munich, Germany). Recombinant standards (98/574 for GH and 02/254 for IGF-I) yielded interassay variability of 4.0–8.7% (IGF-I) and 1.1–3.4% (GH) and sensitivity of 8.8 ng/mL (IGF-I) and 0.04 ng/mL (GH) (9, 10). The limit of quantification for IGFBP3 was 50 ng/mL and the intra- and interassay coefficients of variation (CVs) were 4.2% and 6.9% respectively (11).

#### ALS

Serum ALS levels were measured in duplicate by sandwich immunometric assay using monoclonal antibodies directed against specific N- and C-terminal oligopeptides (12). A serum pool of healthy male volunteers was used for calibration and assigned 1000 U/L. The assay range is 500–5000 U/L, and the intra- and inter-assay CVs were less than 9%. All samples from an individual subject were analysed in one run.

#### GHBP

Serum GHBP levels were measured by a modification of the ligand immunofunctional assay with an in-house monoclonal anti-GHBP antibody. Within-assay CVs were 9.4% at 115 pmol/L and 6.1% at 1550 pmol/L. At the same concentrations, between-assay CVs were 8.5% and 10.9% respectively. The lower limit of quantification was 69 pmol/L, and the linear range was 69–3500 pmol/L. All samples from an individual subject were analysed in one run (13).

### Statistical analysis

The study was powered for within-group comparison of serum IGF-I (primary efficacy variable).

Based on a published pegvisomant study (3), a clinically meaningful reduction in baseline IGF-I was determined to be 27.5%, with a conservative estimate of s.d. of 30%. To achieve a level of significance of 0.05 with a two-sided test, it was determined that a minimum sample size of 12 patients per treatment was required to achieve a power of at least 80%.

The planned efficacy analyses were within the intention-to-treat group. For the primary efficacy variable, the null hypothesis of no percentage change in fasting IGF-I levels from baseline to week 14 was tested for each treatment group with a (two-sided) one-sample *t* test and Wilcoxon signed-rank test. In addition, a pre-specified, though not powered for between-treatment groups, comparison was performed using a Wilcoxon rank-sum test (mathematically equivalent to Mann–Whitney *U* test) for serum IGF-I 1 week after the last dose of study drug (week 14).

Baseline to week 14 testing for both cohorts was undertaken (Wilcoxon signed-rank test) for each of the secondary endpoints (the other components of the IGF ternary complex, namely IGFBP3 and ALS, plus circulating GH and GHBP, SSS, ring size and AcroQoL) and presented as median plus range.

A *post hoc* regression analysis of the relationship between change in IGF-I and dose/kg/week is reported with the associated 95% CI. Comparison of trapezoidal area under the curve for GH during the OGTTs is reported. Statistical significance is indicated by a *P* value <0.05.

Serum IGF-I data are expressed in mass units (ng/mL) and as a percentage of the upper limit of the age-related reference range.

### Data Safety Monitoring Board

An independent Data Safety Monitoring Board (DSMB) was established prior to recruitment start, with an appropriate charter to direct decisions and monitor the trial safety results at intervals throughout the study and provided their recommendations as to whether the trial could continue as planned or whether there were any concerns. The DSMB comprised four individuals with appropriate experience in the areas of acromegaly, endocrine disorders, statistics and the conduct of clinical trials.

## Results

### Participants

**Figure 3:**
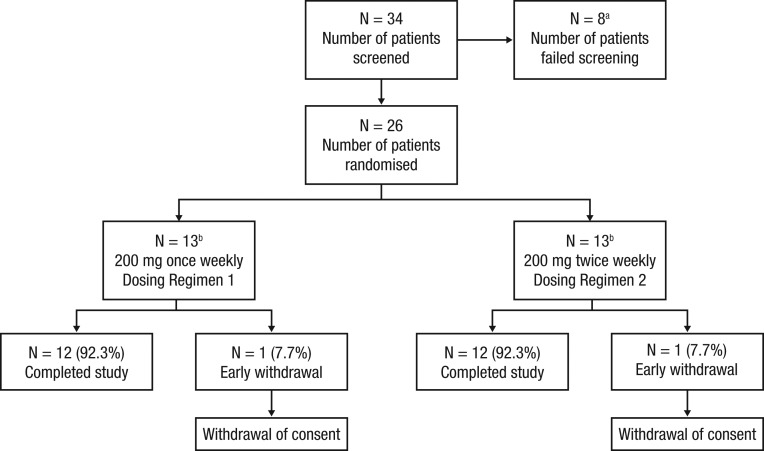
Patient disposition. ^a^Five patients failed screening as IGF-I was <130% ULN. ^b^Although powered for 12 patients per arm, 13 were included per arm since a commitment had been made to allow patients consented and ‘passing’ screening to receive study drug. IGF-I, insulin-like growth factor 1; ULN, upper limit of normal.

Thirteen patients with active acromegaly (IGF-I >130% ULN at screening visit) (detailed in Fig. 3 and Table 1) were recruited into each study arm.

**Table 1:** Baseline characteristics of patients with acromegaly. Data are presented as median (range).

	200 mg ATL1103 once weekly	200 mg ATL1103 twice weekly
Number of patients	13	13
Age (years)	49 (26–72)	49 (32–80)
Sex (M/F)	5/8	6/7
Duration of disease* (years)	9.0 (1–24)	3 (<1–20)
Weight (kg)	90.6 (73.4–113.9)	83.2 (58–131.6)
Height (cm)	169 (154–194)	163 (148–197)
BMI (kg/m^2^)	31.8 (26.0–39.6)	29.4 (21.4–45.3)
Size of adenoma at diagnosis (no. (%))		
Micro (<10 mm)	2 (18)	2 (16.7)
Macro (≥10 mm)	9 (81.8)	10 (83.3)
Missing	2	1
Hypopituitarism at study entry (no. (%))	4 (30.8)	4 (30.8)
Previous therapy (no. (%))		
Surgery	13 (100)	12 (92.3)
Radiotherapy (all modalities)	6 (46.2)	5 (38.5)
Dopamine agonist therapy	3 (23)	5 (38.5)
Somatostatin analogue therapy	11 (84.6)	12 (92.3)
Pegvisomant therapy	7 (53.8)	5 (38.5)
Serum growth hormone (ng/mL)	3.6 (0.4–60.6)	3.5 (1.5–9.4)
GH nadir (screening OGTT) (ng/mL)	2.5 (0.29–54.69)	2.4 (0.37–5.52)
Serum IGF-I (ng/mL)	447 (205–975)	642 (239–831)^†^
Serum GHBP (pM)	1179.0 (386–7637)	525.0 (<69–6434)^‡^
Serum IGFBP3 (ng/mL)	6589.0 (5162–9630)	7005.0 (3396–9843)
ALS (mU/mL)	1669.0 (1395–2829)	1970.0 (945–2463)
Ring size circumference (mm)	63.8 (57.5–81.4)	67.5 (53.7–78.9)
AcroQoL (global score)	58 (18–100)	71 (56–90)
SSS (calculated maximum score)	20.0 (1–36)^§^	11 (7–30)

*Years from initial diagnosis to first day of study; ^†^baseline IGF-I missing for one patient, screening IGF-I value used for calculations; ^‡^*n* = 12, baseline GHBP missing for one patient; ^§^*n* = 12, baseline SSS missing for one patient.

ALS, acid labile subunit; GH, growth hormone; GHBP, growth hormone–binding protein; IGFBP-3, insulin-like growth factor binding protein 3; OGTT, oral glucose tolerance test; IGF-I, insulin-like growth factor 1; SSS, signs and symptoms score.

### Efficacy

#### IGF-I

**Figure 4:**
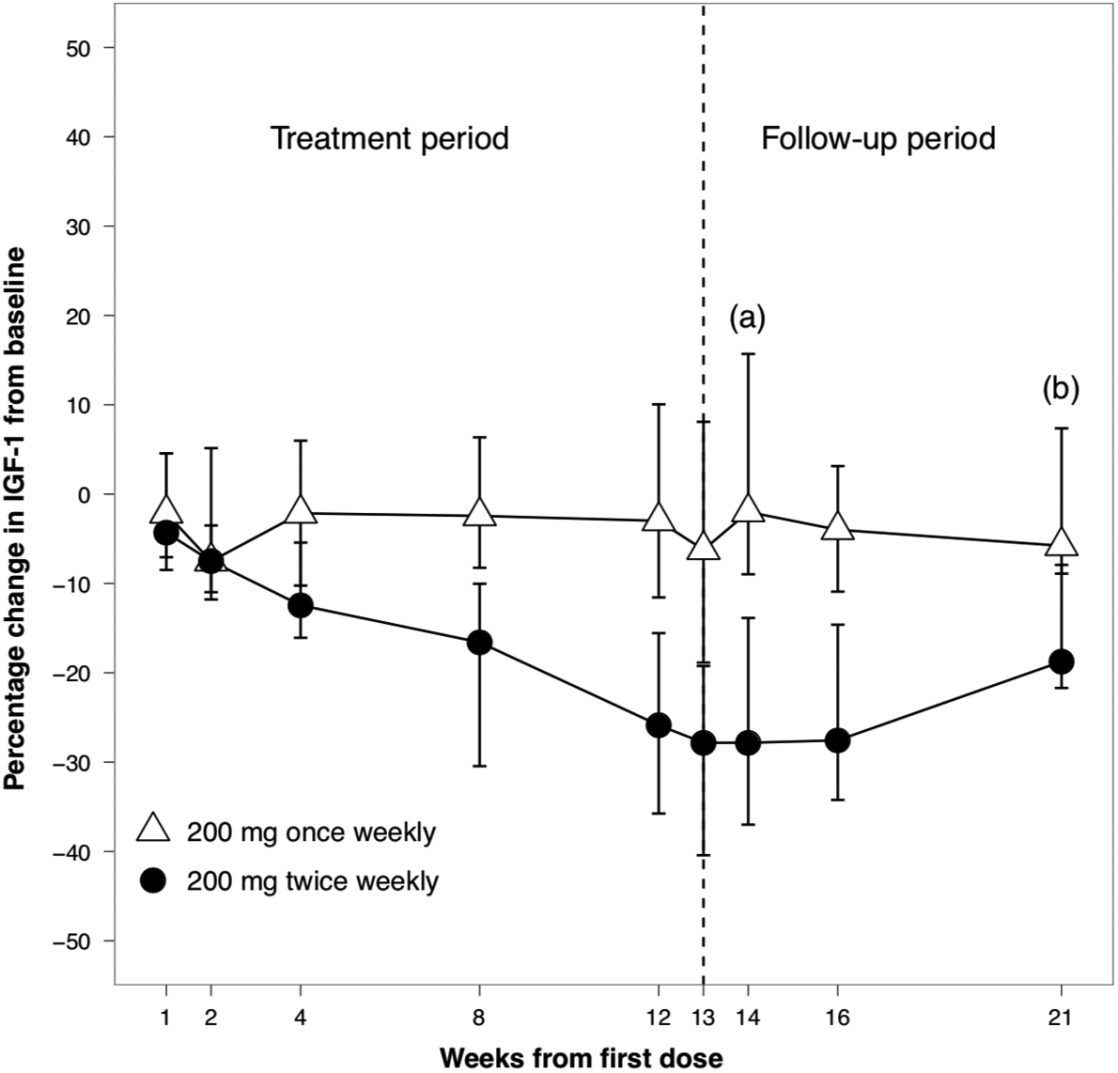
Median percentage change from baseline in serum concentrations of IGF-I in patients with acromegaly. In the patients randomised to ATL1103 200 mg twice weekly, the median fall in serum IGF-I was 27.8% (*P* = 0.0002^a^) at the end of the treatment phase (week 14, 1 week after the last dose of study drug) compared to baseline (week 0). Between-cohort analysis at week 14 demonstrated the median fall in serum IGF-I to be 25.8% (*P* = 0.0012) with twice-weekly compared to once-weekly dosing. In the twice-weekly cohort, IGF-I was still declining at week 14, and at week 21 remained lower than at baseline by a median of 18.7% (*P* = 0.0005^b^). Medians and interquartile ranges are plotted. IGF-I, insulin-like growth factor 1.

**Figure 5:**
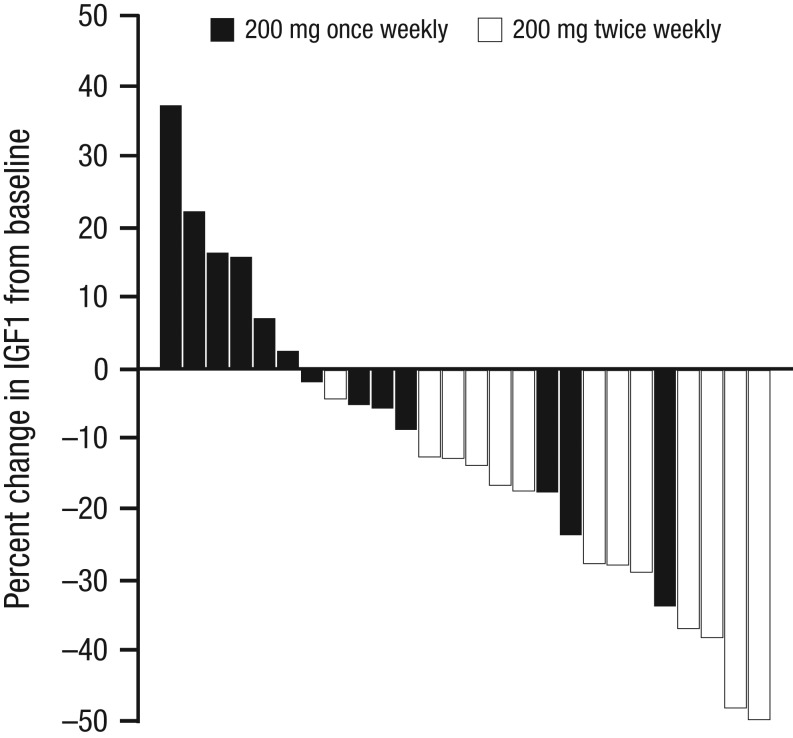
Percentage change in serum IGF-I levels from baseline to week 14 in 26 patients treated with 200 mg ATL 1103 once or twice weekly. IGF-I, insulin-like growth factor-1.

At baseline, the median serum IGF-I was 447 ng/mL (205–975) (2.5 × ULN) and 649 ng/mL (239–831) (2.75 × ULN) ng/mL, in the once- and twice-weekly groups respectively. Compared to baseline, at week 14, ATL1103 at a dose of 200 mg twice weekly resulted in a median fall in serum IGF-I of 27.8% (range 4.4–49.8%, *P* = 0.0002), while no change was seen with once-weekly dosing. At week 14, the median fall in IGF-I was 25.8% greater with twice-weekly compared to once-weekly dosing (*P* = 0.0012). In the twice-weekly cohort, IGF-I levels were still declining at week 14, and at the end of the washout (week 21) remained lower than at baseline by a median of 18.7% (*P* = 0.0005) (Figs 4 and 5).

In both dosing regimens, one patient had an IGF-I within the age-related reference range at the pre-defined endpoint of week 14. Normalisation of IGF-I at any time point was a pre-defined outcome measure and was met by one additional patient in the twice-weekly regimen (week 13).

**Figure 6:**
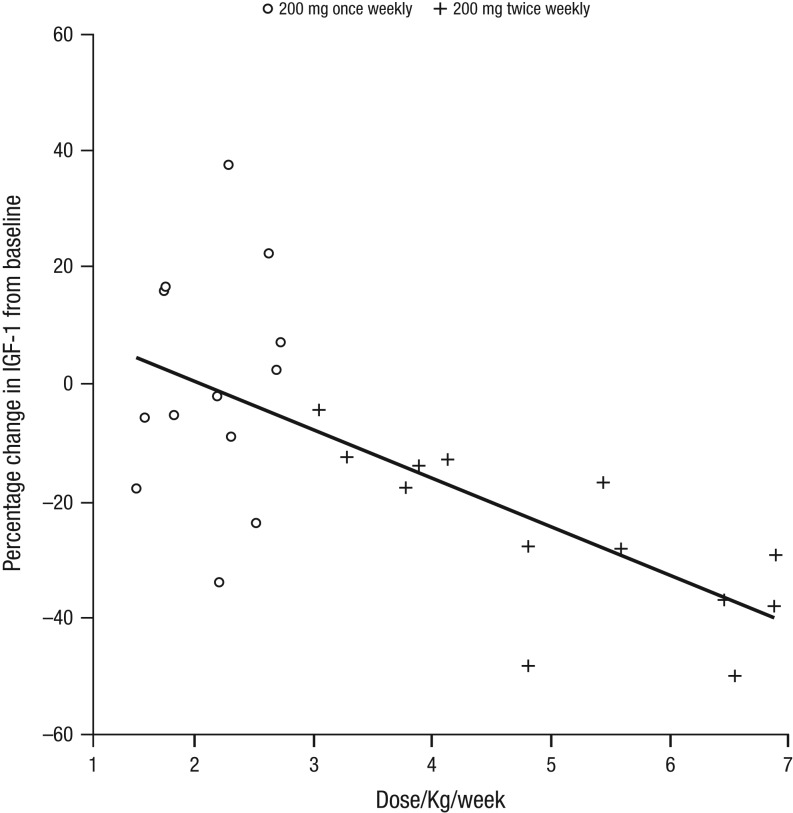
Scatterplot of the percentage change from baseline in IGF-I at week 14 by the allocated dose/kg/week. Combining the data from the two dosing regimens demonstrated a highly statistically significant (*P* = 0.0001) correlation with an estimated slope of regression of −8.27 (95% CI −11.97 to −4.56). IGF-I, insulin-like growth factor 1.

Combining the data from the two dosing regimens, regression analysis of the percentage change in IGF-I levels vs dose/kg/week (median 2.88 (range 1.52–6.90)) demonstrated an estimated slope of regression of −8.27, indicative of a highly statistically significant (*P* = 0.0001) association between fall in IGF-I and the dose/kg/week (Fig. 6).

#### IGFBP3

In the twice-weekly cohort, at week 14, there was a median fall in serum IGFBP3 of 8.9% (range −29.2 to 12.9%, *P* = 0.027) from baseline (median: 7005 ng/mL, range: 3396–9843). Once-weekly ATL1103 did not result in a significant change in serum IGBP3.

#### ALS

Compared to baseline, twice-weekly ATL1103 resulted in a median fall in ALS at week 14 of 16.7% (range −20.9 to 34.9%, *P* = 0.017) from baseline (median: 1970 mU/mL, range: 945–2463). Once-weekly ATL1103 did not result in a significant change in serum ALS.

#### GH

**Figure 7:**
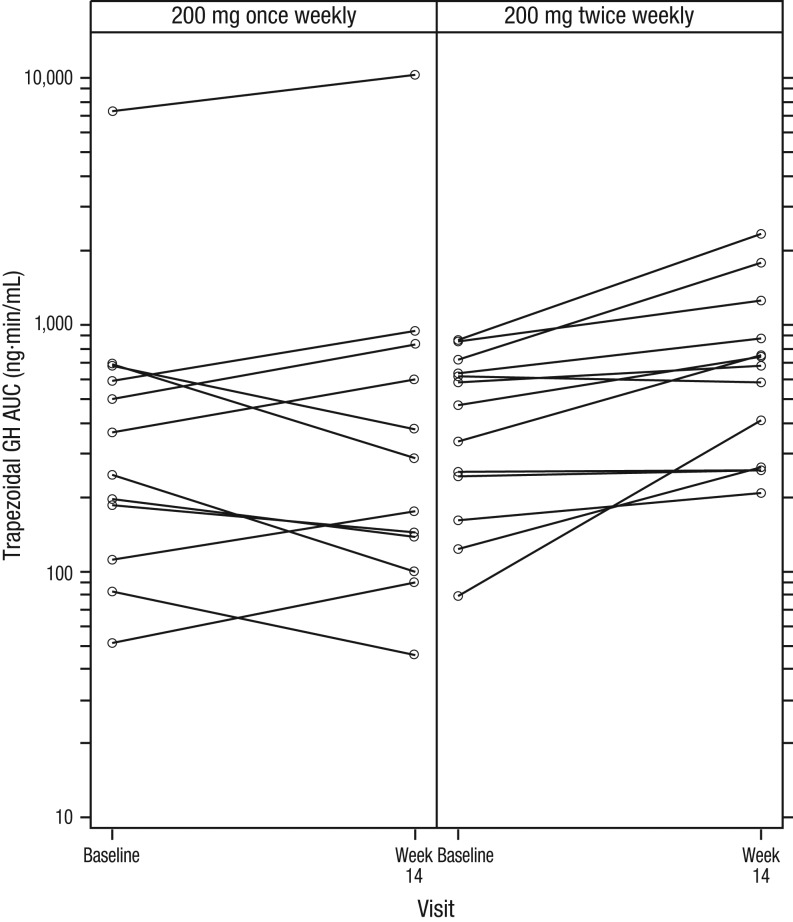
Trapezoidal AUC for GH during OGTTs at baseline and week 14. In the twice-weekly cohort, the median increase in AUC was 46% at week 14 compared to baseline (*P* = 0.001). There was no change in GH levels in the once-weekly cohort. AUC, area under the curve; GH, growth hormone; OGTT, oral glucose tolerance test.

In the twice-weekly cohort, median trapezoidal AUC for GH during the OGTT had increased by 46% (range −5.4 to 419%, *P* = 0.001) at week 14 compared to baseline (471 ng·min/mL (79–867)). There was no change in GH levels in the once-weekly cohort (Fig. 7).

#### GHBP

**Figure 8:**
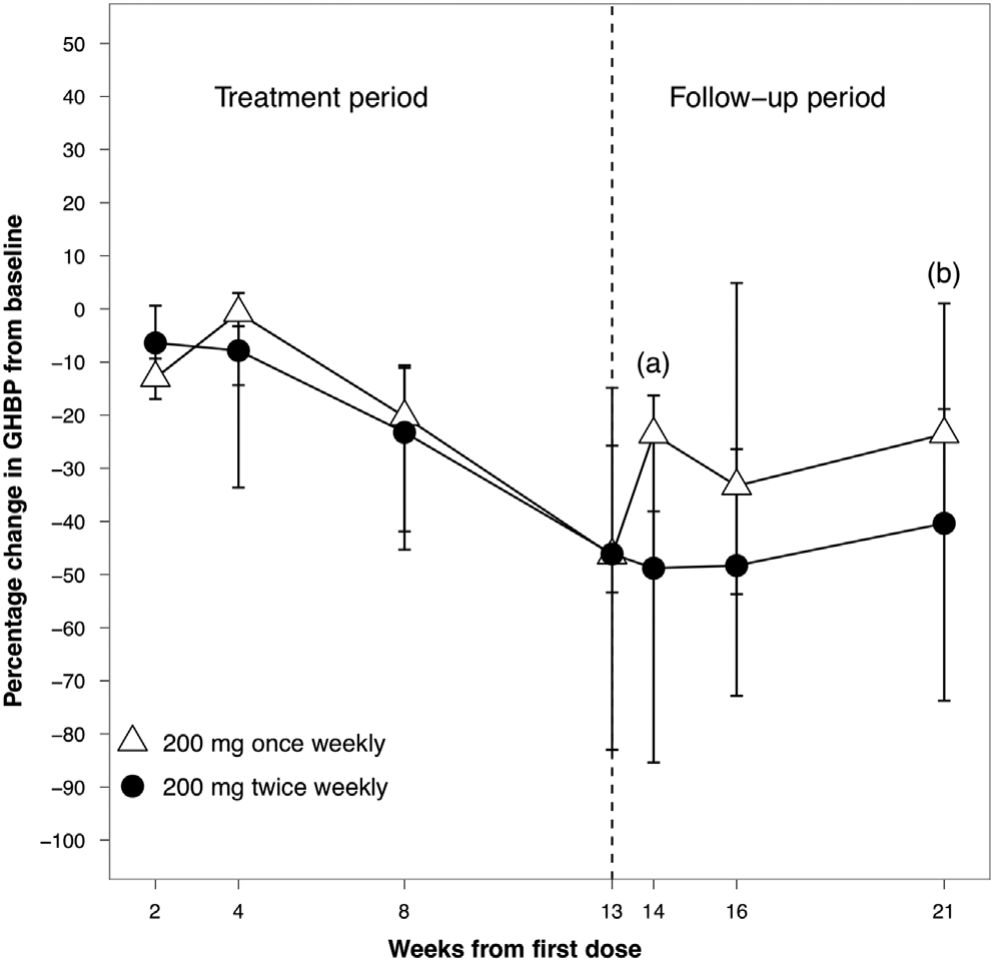
Median percentage change from baseline in serum concentrations of GHBP in patients with acromegaly. Twice-weekly ATL1103 resulted in a median decline of 48.8% in GHBP (*P* = 0.005^a^) at week 14 (open symbols), while a median fall of 23.6% was seen in the once-weekly cohort (*P* = 0.027^a^). In the twice-weekly cohort, at week 21 the median fall in GHBP, compared to baseline, was 40.4% (*P* = 0.008^b^). Medians and interquartile ranges are plotted. GHBP, growth hormone–binding protein.

There was a significant decline in serum GHBP levels in both cohorts at week 14. Twice-weekly ATL1103 resulted in a median decline of 48.8% (range −9.8 to 94.1%, *P* = 0.005) in GHBP from baseline (525 pmol/L (<69–6434), while a median fall of 23.6% (range −61.4 to 59.4%, *P* = 0.027) was seen in the once-weekly cohort (1179 pmol/L (386–7637), *P* = 0.027) and was maintained through to week 21. In the twice-weekly cohort, the median fall in serum GHBP at week 21 was 40.4% (range −94.1 to 6.1%, *P* = 0.008) compared to baseline (Fig. 8).

### Ring size

There was a statistically significant decrease in ring size circumference (mm) from baseline to week 13 for regimen 2, with a median decrease of −1.25 mm (range −12.6 to 3.8, *P* = 0.039). Ring size was unchanged with once-weekly dosing.

### Signs and symptoms score

There was no marked difference in either regimen in median SSS at baseline (20 (1–36) vs 11 (7–30)). The median percentage fall from baseline at week 14 was greater for twice-weekly dosing 37.5% (range −185.7 to 91.7%) compared with 10.2% (range −33.3 to 83.3%) for once-weekly dosing, although the changes were not statistically significant.

### AcroQol

The median absolute improvement in the physical dimension and global scores between baseline and week 14 in the once-weekly cohort were 6.25 (range 0 to 31.3, *P* = 0.002) and 3.4 (range −2.3 to 14.8, *P* = 0.0068) respectively, but these parameters did not change significantly with twice-weekly ATL1103.

In contrast, in the twice-weekly cohort, comparing baseline to week 14, the only significant finding was an improvement in the median absolute change for the appearance subsection of psychological dimension of 10.7 (range −17.9 to 25.0, *P* = 0.035). There was no significant improvement in the once-weekly cohort.

### Safety

ATL1103 was well tolerated with mild-to-moderate injection-site reactions (ISRs) being the most common treatment-emergent AE, affecting 85% of patients in both cohorts (Table 2). Four serious AEs (SAEs) were reported, of which three occurred in a single patient taking the once-daily regimen (acute bronchitis, loss of consciousness while driving and cholecystitis) and one in a patient taking the twice-daily regimen (ear infection), but none were felt to be study drug related; both patients completed the 13 weeks of therapy. Two patients from one centre ‘withdrew consent’ at completion of dosing (weeks 13 and 14) with study drug and thus did not participate in the washout period through week 21.).

**Table 2:** Summary of treatment-emergent adverse events (safety set).*

	200 mg once weekly (*n* = 13)			200 mg twice weekly (*n* = 13)			Total (*N* = 26)		
	*n*	*N*	%	*n*	*N*	%	*n*	*N*	%
TEAEs	98	11	84.6	88	11	84.6	186	22	84.6
Drug-related TEAEs^†^	33	6	46.2	24	8	61.5	57	14	53.8
Serious TEAEs	3	1	7.7	1	1	7.7	4	2	7.7
Severe drug-related TEAEs	0	0		0	0		0	0	
Severe TEAEs	6	3	23.1	1	1	7.7	7	4	15.4
TEAEs leading to permanent discontinuation of study drug	0	0		0	0		0	0	
Withdrawals	1^§^				1^§^				
Patients with ISR	11		84.6	11		84.6	22		
Mild		9			6			15	
Moderate		2			5			7	
Severe		0			0			0	
Most frequent TEAEs with a >15% incidence									
Headache	21	4	30.8	5	3	23.1	26	7	26.9
Fatigue	3	2	15.4	3	2	15.4	6	4	15.4
Diarrhoea	3	2	15.4	2	2	15.4	5	4	15.4
Constipation	2	2	15.4	2	2	15.4	4	4	15.4
Dizziness	1	1	7.7	4	2	15.4	5	3	11.5
Hyperhidrosis	1	1	7.7	4	2	15.4	5	3	11.5
Rash	3	1	7.7	2	2	15.4	5	3	11.5
Abdominal pain upper	1	1	7.7	2	2	15.4	3	3	11.5
Nasopharyngitis	2	2	15.4	1	1	7.7	3	3	11.5
Urinary tract infection	3	2	15.4	0	0		3	2	7.7
Oropharyngeal pain	0	0		3	2	15.4	3	2	7.7
Abdominal distension	0	0		2	2	15.4	2	2	7.7
Abdominal pain	0	0		2	2	15.4	2	2	7.7
Tracheitis	2	2	15.4	0	0		2	2	7.7
Carpal tunnel syndrome	2	2	15.4	0	0		2	2	7.7
Haematuria	2	2	15.4	0	0		2	2	7.7
Lipohypertrophy	2	2	15.4	0	0		2	2	7.7

*Excludes ISRs; ^†^drug related is defined as relationship to study drug = possible, probable, or definite; ^§^withdrew consent after last drug dose.

ISR, injection-site reaction; *N*, number of patients; *n*, number of events; %, percentage of patients; TEAE, treatment-emergent adverse event.

One patient in each regimen had low circulating platelet levels at a single time point (weeks 4 and 13, 86 and 132 × 10^9^/L respectively; normal range 150 × 10^9^/L), but these resolved either spontaneously or after treatment end (week 13). Two patients had elevated liver enzymes judged clinically significant: one patient taking the once-daily regimen had γ-glutamyltransferase, AST, ALT and ALP values above the normal limit (>ULN); one patient taking the twice-daily regimen had AST and ALT values >ULN. All effects on liver function were transient (Table 3).

**Table 3:** Summary of abnormal liver function tests.

Analyte	Week	Result	Reference range
200 mg once weekly			
↑ GGT (U/L)	8	159 (repeats: 65, 32)	8–61
	21	102	
↑ AST (U/L)	8	111 (repeats: 70, 26)	6–40
↑ ALT (U/L)	8	181 (repeat: 135)	6–40
↑ ALP (U/L)	8	200	40–128
200 mg twice weekly			
↑ AST (U/L)	4	42	2–31
	8	43	
↑ ALT (U/L)	4	69	8–34
	8	99	
	13	52	
↑ Total bilirubin (μmol/L)	1	28	0–21
	2	22	
	4	26	

ALP, alkaline phosphatase; ALT, alanine aminotransferase; AST, aspartate aminotransferase; GGT, gamma glutamyl transferase.

The treatment-emergent AE (TEAE) profile was comparable for the two treatment groups (Table 2). Almost all patients experienced ISRs (mild and moderate), and ‘mild’ lipohypertrophy, that subsequently, resolved was reported in two patients. There was a greater incidence of headache in the once-weekly vs twice-weekly regimen (21 events and 5 events, respectively), but the number of patients who experienced headache was comparable (four patients in the once-daily regimen compared with three patients in the twice-daily regimen).

Radiologically significant tumour diameter changes (2 mm or more in any one dimension or tumour volume changes of more than 20%) were reported in three patients. Tumour volume increased in two patients (one in each dosing regimen, 5.7 × 7.3 × 19.1 vs 6.8 × 9.9 × 19.5 mm and 8.1 × 5.8 × 14.8 vs 8.1 × 7.2 × 16.2 mm) and reduced in one patient on twice-weekly dosing (6.2 × 10.4 × 4.9 vs 2.6 × 5.7 × 4.1 mm). The changes were judged not to be clinically significant.

## Discussion

The technology underpinning ASO therapy is rapidly advancing and has the potential to offer new therapeutic options across a broad spectrum of diseases. Disordered protein production or function is implicated in most pathological processes, and ‘gene silencing or activating’ by single-stranded ASOs against target RNA sequences is an attractive concept that permits greater specificity than can be achieved with small molecules or antibodies (14). The synthetic structural modifications, such as the phosphorothioate backbone and 2′-O-methoxyethyl modifications, can be readily applied to whole classes of ASOs with only the nucleotide sequence being indication specific. Encouraging studies of ASOs are being reported against many targets, but this is the first report of the use of an ASO in endocrinology.

The data presented provide the ‘proof of concept’ that in patients with acromegaly, an ASO targeting the GHR can lower serum IGF-I and raise the prospect of a new and entirely novel therapy for acromegaly. Thirteen weeks of ATL1103 at a dose of 200 mg twice weekly lowered median serum IGF-I by 27.8%, with two (15%) of 13 patients achieving an IGF-I within the reference range. Serum IGF-I levels were still declining at week 14 and had not returned to baseline by the end of the 8-week (week 21) washout period (Fig. 4), suggesting that the duration of ATL1103 therapy was too short to see maximum benefit and that prolonged treatment at the same doses may result in a further decline in serum IGF-I. First-order drug kinetics indicate that approximately four to five elimination half-lives are required to achieve steady-state plasma concentrations; since the tissue half-life of ATL1103 is believed to be >4 weeks (15), this would suggest that between 16 and 20 weeks of treatment would be required for nadir IGF-I levels to be achieved.

In conjunction with the data indicating a relationship between the dose/kg/week and the fall in serum IGF-I, it seems probable that larger doses of ATL1103 administered for longer are likely to result in a greater fall in IGF-I and offer the prospect of ‘normalisation’ of IGF-I in a greater proportion of patients. Reassuringly, the decline in serum IGF-I with twice-weekly treatment was paralleled by falls in the other elements of the IGF ternary complex, namely IGFBP3 and ALS.

Circulating GHBP is the product of cleavage of the extracellular component of the GH receptor (16). In acromegaly, there is a negative correlation between serum GHBP concentrations and IGF-I and GH levels (17), such that GHBP concentrations are decreased in active acromegaly, and increase with conventional therapy. In contrast, the reduction in IGF-I caused by ATL1103 therapy is associated with a significant decline in serum GHBP concentrations, which were still falling at week 14 and had not returned to baseline by the end of washout at week 21. The fall in GHBP likely reflects the ATL1103-induced downregulation of GHR cell surface number, with a dose–response observed, as the fall was greater with the twice-weekly compared to once-weekly regimen, 23.6% and 48.8% respectively. The changes in GHBP emphasise the difference in action of ATL1103 and future studies with increased doses of ATL1103, and larger cohorts will permit exploration of the relationship between the change in serum IGF-I and circulating GHBP concentrations.

As with pegvisomant, the reduction in serum IGF-I with twice-weekly ATL1103 was associated with a 46% increase in serum GH levels assessed during an OGTT. Studies with pegvisomant have demonstrated that the increased GH secretion is a consequence of negative feedback in response to the fall in circulating IGF-I induced by blocking GH action (18).

It is encouraging that an improvement in the soft-tissue manifestations of acromegaly, indicated by the reduction in ring size, was seen with twice-weekly therapy. The short duration of therapy and small cohort size means it should not be a surprise that the fall in IGF-I was not associated with an improvement in SSS and only very modest improvements in quality of life as measured by AcroQol. Studies in larger numbers of patients treated for longer are required to demonstrate the impact of ATL1103 on well-being and quality of life.

Almost all patients, approximately 85%, experienced ISRs, but otherwise ATL1103 was generally well tolerated with no apparent drug-related SAEs. There were four SAEs, of which three occurred in one patient, and all were judged as unlikely to be drug related; both patients completed the 13 weeks of therapy (Table 2). ISRs are a recognised side effect of second-generation ASOs, were mild to moderate in severity (predominantly a mixture of erythema, pain and pruritus) and affected both cohorts equally. No patient withdrew from this study because of ISRs. This is a similar incidence of ISRs as reported in other studies: 90% of patients participating in a phase 2 study of mipomersen experienced mild-to-moderate ISRs (19). The mechanism of oligonucleotide-induced ISR is yet to be fully elucidated, but skin biopsies in 9 of 32 subjects participating in a phase 1 study of mipomersen were consistent with leukocytoclastic vasculitis (e.g., infiltrating neutrophils, prominent nuclear dust, lymphocytes and eosinophils with local macrophage infiltration) (20). The lessons learned from the numerous other ASOs under clinical development will inform the management of ISRs in future studies of ATL1103. Transient changes in platelet count and liver function were encountered but were judged not to necessitate any change in therapy. Studies of greater length involving larger numbers of patients are required before any conclusions can be drawn about the safety profile of ATL1103.

There were no clinically significant changes in pituitary tumour size, but the short duration of treatment precludes meaningful conclusions about the long-term impact of ATL1103 on this parameter. Both the patients in whom tumour expansion of >2 mm was documented had discontinued somatostatin analogue therapy (one octreotide, one lanreotide) prior to commencing ATL1103, raising the possibility of rebound expansion from somatostatin analogue-induced tumour shrinkage. Reassuringly, the experience from more than 10-year use of pegvisomant is that GHR-targeted therapy does not induce growth of pituitary adenomas (21).

In summary, ATL1103 lowers IGF-I in acromegaly with biochemical changes consistent with downregulation of the GHR. As IGF-I was still declining at the end of the treatment period and with the knowledge that the dose/kg could be increased, ATL1103 has the potential to achieve disease control in a significant proportion of patients. Placebo-controlled studies of longer duration and using higher doses are needed to better assess the full potential of this novel treatment.

## Declaration of interest

PJT received research support from Antisense Therapeutics during the conduct of the study and with presenting the results. He has served on advisory boards (unpaid) and/or received research support from for Novartis, Ipsen, Chiasma, Strongbridge and Ono. J D C N-P served as a consultant and steering committee member and received research support from Novartis. P C received research support from Antisense Therapeutics, Novartis, Ipsen, Pfizer and Italfarmaco. T B received personal fees from Antisense Therapeutics and received research support and personal fees from Pfizer, Ipsen and Novartis. J A served as a speaker and on advisory boards for Pfizer, Ipsen and Novartis. G T and L A are employees of Antisense Therapeutics and own stock. M B received research support from Antisense Therapeutics, research support and personal fees from Chiasma and Novartis and personal fees from ONO. All other authors have no relevant disclosures.

## Funding

The study was funded by Antisense Therapeutics Limited (Melbourne, Australia).

## Author contribution statement

P J T contributed to the study design, identification of participating sites and review of study data and drafted the manuscript. J D C N-P participated in patient recruitment, contributed to drafting the manuscript and reviewed and approved the manuscript. J A, S J B A, A R, W D, P C, T B, S M W, C F, J A, A I M and D J T participated in patient recruitment and reviewed and approved the manuscript. G T and L A were involved in protocol development and reviewed and approved the manuscript. D R was responsible for the data analysis contained in the manuscript. M B contributed to the study design and measurement of IGF-I, GH, IGFBP3 and ALS and reviewed and approved the manuscript.
